# Exploring the Impact of Security Technologies on Mental Health: A Comprehensive Review

**DOI:** 10.7759/cureus.53664

**Published:** 2024-02-05

**Authors:** Adwait S Malik, Sourya Acharya, Sonal Humane

**Affiliations:** 1 Community Medicine, Jawaharlal Nehru Medical College, Datta Meghe Institute of Higher Education & Research, Wardha, IND; 2 Medicine, Jawaharlal Nehru Medical College, Datta Meghe Institute of Higher Education & Research, Wardha, IND; 3 Department of Mental Health Nursing, Srimati Radhikabai Meghe Memorial College of Nursing, Datta Meghe Institute of Higher Education & Research, Wardha, IND

**Keywords:** privacy protection, surveillance, ethical guidelines, user perception, mental health, security technologies

## Abstract

This comprehensive review explores the intricate relationship between security technologies and mental health. Security technologies, including physical security, cybersecurity, and surveillance measures, are integral components of our modern world, designed to protect individuals, organizations, and society from various threats. While they are vital in enhancing safety, they also have profound implications for mental well-being. The review delves into the positive impacts of security technologies, including their capacity to enhance personal safety, reduce anxiety and fear, and instill a sense of security. However, it also reveals the negative consequences, such as privacy invasion, surveillance-related stress, paranoia, and ethical concerns, which can erode mental health. User perception and trust are central to understanding how individuals experience security technologies. The review emphasizes the importance of ethical guidelines, user education, and technological advancements in mitigating negative impacts. By embracing an ethical-by-design approach, empowering users, and promoting public awareness, a balanced equilibrium between security and mental health can be achieved. The conclusion highlights the significance of ongoing research and interdisciplinary collaboration to navigate this intricate relationship effectively. By prioritizing ethical considerations and fostering a dialogue that values security and individual well-being, we can ensure a safer and more mentally healthy future in our technologically interconnected world.

## Introduction and background

In our rapidly evolving technological landscape, security technologies have become an integral part of our daily lives. From surveillance cameras in public spaces to firewalls guarding our online identities, these tools play a vital role in safeguarding our physical and digital worlds. While the primary purpose of security technologies is to protect us from potential threats, it is essential to recognize that their presence and use can have significant implications for our mental well-being [1]. The deployment of security technologies has grown exponentially in recent years, partly driven by concerns related to public safety, terrorism, cyberattacks, and data breaches. This pervasive integration of security measures into our surroundings has sparked a growing interest in understanding the broader consequences of these technologies on individuals' mental health. Consequently, it is imperative to delve deeper into this complex relationship, examining both the positive and negative aspects it entails [2].

The importance of this topic lies in its potential to shed light on a multifaceted issue that affects millions of individuals worldwide. The impact of security technologies on mental health touches on fundamental aspects of human existence, including the right to privacy, personal security, and the balance between security and personal freedom. As society grapples with the challenges posed by an increasingly digitized and surveilled world, understanding the psychological implications of these technologies becomes crucial for policymakers, technologists, mental health professionals, and the public [3]. Moreover, in the wake of unprecedented technological advancements, the intersection of security technologies and mental health presents opportunities and challenges. By comprehensively examining this issue, we can identify best practices, formulate ethical guidelines, and create a nuanced understanding of how these technologies can coexist with individuals' well-being [4].

The primary objective of this comprehensive review is to delve deeply into the influence of security technologies on mental health. By consolidating existing research, case studies, and expert insights, we aim to accomplish several essential goals. Firstly, we seek to evaluate the positive impacts of security technologies on mental well-being, which encompass aspects like improved personal safety and a heightened sense of peace. Second, our focus extends to examining the negative consequences, encompassing concerns linked to privacy infringements, stress induced by surveillance, and ethical quandaries. Additionally, we will explore the pivotal role of user perception in shaping mental health outcomes within security technologies. Furthermore, our review aims to pinpoint potential strategies and technologies to alleviate the adverse effects and promote responsible utilization of security technologies. Finally, we aspire to provide valuable insights and recommendations that can inform future research, policy development, and public discourse concerning the critical intersection of security and mental health.

## Review

Security technologies and mental health

Overview of Security Technologies

Physical security: Physical security measures constitute a critical aspect of safeguarding tangible, real-world assets and spaces. These technologies encompass a variety of tools and systems that aim to prevent unauthorized access, intrusions, and breaches in physical locations. Access control systems, for instance, regulate entry to buildings or specific areas within them, ensuring that only authorized individuals can enter. Alarm systems are designed to detect and notify of breaches or unauthorized activities, triggering responses to mitigate potential threats. Video surveillance utilizes cameras to monitor physical spaces, offering both real-time monitoring and recorded footage for investigative purposes. Biometrics, such as fingerprint or retina scans, provide a highly secure method for verifying the identity of individuals, often used in high-security environments. Security personnel, including guards and security officers, play a human role in maintaining physical security, offering on-the-ground surveillance and immediate response to security incidents [5].

Cybersecurity: Unlike physical security, cybersecurity is primarily concerned with safeguarding digital assets, data, and network infrastructure. With the ever-increasing reliance on digital technology, cybersecurity technologies are crucial in protecting against various digital threats. Critical components of cybersecurity include firewalls, which serve as digital barriers to prevent unauthorized network access and protect against cyberattacks. Antivirus software is designed to detect and remove malicious software, or malware, that can compromise digital systems. Encryption is a technique to secure data and communications by converting it into a code that can only be deciphered by authorized parties. Intrusion detection systems monitor network traffic for signs of potential threats and can trigger alarms or responses to mitigate cyberattacks. In essence, cybersecurity measures are vital for safeguarding sensitive information and the functionality of digital systems [6].

Surveillance: Surveillance technologies encompass various tools and systems used for monitoring and tracking activities, often in both public and private settings. Closed-circuit television (CCTV) cameras are a standard surveillance technology widely used in public areas, commercial spaces, and residential settings. They capture video footage that can be observed in real time or recorded for later analysis. Facial recognition software is an advanced form of surveillance that uses biometric data to identify individuals based on their facial features. Surveillance serves various purposes, including monitoring public spaces for security and law enforcement, securing private property, and tracking individuals for identification and security purposes. The potential applications of surveillance technologies are broad. Still, they also raise significant privacy and ethical considerations, as they have the power to capture and analyze vast amounts of personal data and information [7].

Types of Security Technologies

Physical security technologies: This category encompasses a range of tangible measures and systems designed to safeguard physical spaces and assets. Access control systems are instrumental in regulating who can enter specific areas, such as secured buildings or restricted zones. These systems can involve keycards, biometric scanners, or PIN codes, ensuring that only authorized individuals gain access. Security alarms serve as vigilant guardians, promptly notifying individuals or authorities of unauthorized entry or security breaches, effectively deterring potential intruders. Security guards are human elements within physical security, providing on-site monitoring and immediate responses to security incidents. These professionals are often visible deterrents and a reassuring presence in spaces such as malls, airports, and corporate offices. Perimeter defenses include barriers and protective measures surrounding an area to prevent unauthorized entry. These defenses may include fences, walls, security lighting, or advanced technologies like motion sensors [8].

Cybersecurity technologies: In the digital age, cybersecurity technologies protect the vast data, networks, and digital systems we rely on. As technology advances, so do the threats it faces, necessitating an evolving arsenal of protective tools. Firewalls serve as digital barriers, preventing unauthorized access to networks and systems filtering incoming and outgoing traffic for potential threats. Antivirus software is dedicated to identifying and eliminating malicious software or malware that could compromise the integrity of digital systems. Encryption, a fundamental cybersecurity technique, secures data and communications by converting information into code, which can only be deciphered by authorized parties. Intrusion detection systems are integral for monitoring network traffic and identifying signs of potential threats or attacks. These systems, through automated processes or human intervention, can trigger alarms and initiate responses to mitigate cyberattacks. Cybersecurity technologies are paramount for preventing data breaches, protecting sensitive information, and ensuring the continued functioning of digital infrastructure [1].

Surveillance technologies: Surveillance technologies encompass various tools and systems for monitoring and tracking activities in various contexts. Video surveillance is one of the most recognizable forms, using cameras to capture real-time footage or recorded video for later analysis. These cameras are ubiquitous in both public and private settings, serving purposes such as security and crime prevention. Biometrics, a more advanced form of surveillance, employs unique biological characteristics like fingerprints or retinas for individual identification and security purposes. Facial recognition technology is another facet, employing facial features for identification and authentication, often utilized in security, access control, and personal devices like smartphones. While surveillance technologies are designed to enhance security, they also introduce complex concerns regarding privacy and personal autonomy. The presence of surveillance cameras in public spaces and their potential for continuous monitoring can lead to debates and discussions about civil liberties and data protection. Striking the right balance between security and privacy remains an ongoing challenge in surveillance technologies [9].

Overview of Mental Health and Its Importance

Personal well-being: Good mental health is foundational for personal well-being. It encompasses emotional and psychological aspects of an individual's life and contributes to self-esteem, resilience, and ability to navigate life's challenges effectively. When mental health is positive, individuals are more likely to have a positive self-perception and an overall sense of contentment. They can manage stress, handle setbacks, and maintain a healthy perspective on life's ups and downs. It allows individuals to find joy and fulfillment in their daily experiences and relationships [10].

Social functioning: Mental health is intricately linked to an individual's capacity to form and maintain healthy relationships, both in personal and professional contexts. It provides the emotional foundation for effective communication, empathy, and the ability to connect with others on an authentic level. People with good mental health are better equipped to navigate the complexities of human interaction, resolve conflicts, and build meaningful, supportive relationships. This, in turn, enhances their social functioning and overall quality of life [11].

Productivity and creativity: A sound mental state catalyzes productivity and creativity. It enables individuals to think critically, solve problems, and approach tasks with innovation and enthusiasm. People with good mental health tend to be more adaptable, open to new ideas, and capable of finding creative solutions to challenges. Productivity and creativity extend to various aspects of life, from professional work to hobbies and interests, contributing to personal growth and achievement [12].

Physical health: Mental health is closely interconnected with physical health, and the mind-body connection is well-established. Psychological stress, anxiety, and mental health issues can have adverse effects on physical health. Prolonged stress, for example, can contribute to chronic illnesses, such as cardiovascular diseases, digestive problems, and weakened immune function. Conversely, positive mental health can lead to healthier behaviors, such as regular exercise and balanced nutrition, which promote overall physical well-being [13].

Societal impact: Mental health is not merely an individual concern; it carries significant societal implications. Widespread mental health issues can strain healthcare systems and resources, impacting the availability and quality of mental healthcare. In workplaces, mental health plays a critical role in productivity and employee well-being. When mental health issues go unaddressed, they can lead to absenteeism, reduced productivity, and increased healthcare costs for employers. On a broader societal scale, untreated mental health conditions can contribute to social challenges, such as homelessness, addiction, and crime. Addressing mental health at both the individual and societal levels is essential for promoting overall well-being and preventing the potential ripple effects of untreated mental health issues [14].

Positive impacts of security technologies on mental health

Enhancing Personal Safety

Crime deterrence: Security technologies are pivotal in deterring potential criminals and reducing the likelihood of criminal incidents. Surveillance cameras, alarms, and access control systems act as visible deterrents, signaling to potential wrongdoers that a location is monitored and secured. This visibility creates a psychological barrier, dissuading individuals with criminal intentions from engaging in unlawful activities. As a result, these security measures can contribute to a heightened sense of personal safety. Communities and individuals residing in areas equipped with such technologies often experience reduced crime rates, fostering a safer environment where people can live and work without constant fear of criminal activity [15].

Emergency response: Many security technologies are seamlessly integrated with emergency response systems, ensuring that help is readily available during a security breach or threat. When a breach occurs, these systems can swiftly trigger alarms, notify security personnel, and alert law enforcement or emergency services. This immediate response provides individuals with a reassuring sense of security, knowing that assistance is just a call or alarm away. The rapid reaction to security incidents not only mitigates potential harm but also bolsters individuals' confidence that their well-being is a priority, whether in a residential or business setting. This assurance significantly contributes to a greater sense of overall security [16].

Safety in public spaces: Deploying security technologies in public spaces, such as parks, streets, and transportation hubs, enhances the safety of citizens. Surveillance cameras, in particular, offer constant monitoring, creating a visual record of activities and deterring criminal behavior. In well-monitored public areas, individuals often experience an increased sense of safety, as the presence of these security measures fosters an environment where unlawful activities are less likely to occur. This sense of security encourages community members to engage more actively in public life, such as attending public events, using public transportation, and participating in recreational activities without fearing crime. Consequently, security technologies help promote community cohesion and the active use of shared spaces [17].

Reducing Anxiety and Fear

Home security: Residential security systems, including alarms, surveillance cameras, and smart locks, offer homeowners a heightened sense of security and peace of mind. These technologies act as a robust deterrent against home intrusion and unauthorized access. Knowing that their homes are equipped with these security measures makes residents feel more secure. This increased sense of safety extends beyond the physical aspect; it also alleviates psychological stress and anxiety associated with concerns about potential break-ins or trespassing. The knowledge that their homes are protected contributes significantly to residents' mental well-being, enabling them to enjoy a greater sense of comfort and tranquility in their everyday lives [18].

Workplace security: Security measures in workplaces, such as access control systems, surveillance cameras, and alarm systems, have a profound impact on employee well-being. Employees who feel safe and secure in their workplace will likely experience lower stress levels and greater job satisfaction. Knowing that the workplace is equipped with these security technologies instills confidence in employees, enabling them to focus on their work without undue concern about unauthorized access, theft, or other security-related issues. The result is an improved work environment where individuals can dedicate their energy and attention to tasks, fostering a more positive and productive atmosphere [19].

School and educational facilities: Security technologies in educational settings serve a dual purpose by enhancing safety for students and staff while promoting a conducive learning environment. In an era where safety concerns in schools are prevalent, these technologies offer reassurance to students and their families. The knowledge that schools are equipped with security measures, such as surveillance cameras, access control systems, and emergency response protocols, alleviates fear and anxiety. As a result, students can focus on their studies with a reduced sense of vulnerability, leading to better educational outcomes and improved mental well-being. Furthermore, staff members can carry out their roles more effectively, knowing that their workplaces are secure, contributing to their job satisfaction and overall well-being. In essence, security technologies are pivotal in creating an environment where students and educators can thrive with peace of mind [20].

Providing a Sense of Security

Routine and predictability: The presence of security measures establishes a sense of routine and predictability in individuals' lives. Knowing these technologies are in place creates a structured environment that individuals can rely on. This predictability can have a profound impact on mental well-being by reducing the element of surprise associated with potential threats. When individuals are aware that security measures are consistently operational, they can navigate their daily lives with a greater sense of ease and reduced anxiety. This routine fosters a feeling of control and order, which can be especially valuable in uncertain or potentially risky situations. Predictability and routine are comforting aspects that contribute to overall mental stability and well-being [21].

Peace of mind: In an increasingly digital world, cybersecurity measures are vital in providing peace of mind. Knowing that one's personal information and digital assets are protected from cyber threats and unauthorized access is a source of reassurance. This sense of security in the virtual realm is essential for overall mental well-being. It enables individuals to engage in online activities, such as e-commerce, social networking, and digital communication, with a reduced fear of data breaches or cyberattacks. This peace of mind extends to both personal and professional aspects of life, allowing individuals to conduct their online activities without the constant worry of potential digital threats. As a result, they can focus on the positive aspects of the digital world, enhancing their overall mental well-being [22].

Community and social confidence: Communities and social groups that visibly employ security technologies often report increased confidence and cohesion among their members. Knowing these technologies are in place fosters a collective sense of security and trust among community members. The visibility of security measures, such as surveillance cameras in public spaces or neighborhood watch programs, creates a shared understanding that safety is a priority. This sense of collective security can increase social confidence and cohesiveness within the community. Individuals feel more secure and trust their neighbors and community institutions, which can have a positive impact on social interactions, community engagement, and overall well-being. This collective confidence is not only reassuring but also contributes to a harmonious and supportive social environment [23].

Negative impacts of security technologies on mental health

Invasion of Privacy

Constant monitoring: Surveillance technologies, particularly those involving extensive data collection and continuous monitoring, can create a pervasive sense of constant scrutiny. Individuals may feel they are being watched or observed at all times, even in their most private moments. This perception of constant surveillance can erode an individual's sense of personal privacy, leading to feelings of unease, stress, and discomfort. The awareness of being monitored may limit a person's ability to relax and be themselves, as they may constantly second-guess their actions and behaviors, worried that they are being scrutinized. This heightened state of alertness and self-consciousness can take a toll on mental well-being and may even lead to heightened stress and anxiety [24].

Data privacy concerns: In the digital realm, cybersecurity measures often necessitate the collection and storage of personal information for authentication and protection purposes. However, this data collection can raise significant concerns about data privacy. The fear of data breaches, unauthorized access, or misuse of personal data can lead to heightened anxiety and mistrust. Individuals may worry about the security of their digital identities, financial information, and personal communications. This fear can result in individuals being reluctant to engage in online activities, such as e-commerce or social networking, that require the sharing of personal information. The pervasive concerns about data privacy in the digital age can contribute to a sense of vulnerability and a diminished sense of well-being [25].

Fear of surveillance: The awareness of being under surveillance, even in public spaces, can instill a sense of vulnerability and self-censorship. Individuals may hesitate to express themselves freely or engage in activities they would otherwise enjoy. The fear of being watched can lead to self-imposed restrictions on one's behavior and speech, as people may need to conform to perceived norms or expectations. This fear of surveillance can limit personal freedoms and the ability to engage in open and candid discussions. In such an environment, individuals may refrain from expressing their opinions or engaging in activities they cherish, which can have a detrimental impact on their mental well-being, as it may lead to feelings of constraint and a reduced sense of personal freedom [26].

Surveillance-Related Stress

Heightened anxiety: The awareness of being under surveillance can induce heightened anxiety in individuals. The constant knowledge that their actions, whether in public or private settings, are subject to scrutiny can lead to a pervasive sense of vulnerability. People may feel exposed and self-conscious, fearing their behavior is being observed and evaluated. This heightened anxiety is not limited to specific situations but can become a persistent emotional state. It may make individuals increasingly guarded, making it difficult to relax and be themselves. Over time, this sustained anxiety can lead to stress and negatively impact overall mental well-being [27].

Self-censorship: The fear of surveillance often leads to self-censorship, where individuals modify their behavior, communication, or actions to conform to perceived societal or surveillance norms. This self-censorship can manifest in various ways, from withholding personal opinions and engaging in self-expression to avoiding certain activities or discussions. Individuals may refrain from expressing dissenting opinions, engaging in creative or unconventional pursuits, or participating in activities they enjoy. The constant need to conform to perceived expectations can limit personal expression and hinder individual well-being. Over time, self-censorship can erode a person's sense of freedom and authenticity, leading to a less satisfying and fulfilling life [28].

Impact on mental health disorders: Surveillance-related stress can exacerbate pre-existing mental health disorders, particularly conditions such as anxiety and paranoia. Individuals who are already struggling with these disorders may experience heightened symptoms when placed in environments with extensive surveillance. The awareness of being watched can intensify existing distrust, paranoia, and fear. This heightened emotional distress can lead to a deterioration of mental health, with individuals experiencing increased anxiety, panic attacks, and intrusive thoughts. Moreover, the persistent sense of surveillance can create a hostile environment for those with pre-existing mental health conditions, making it challenging for them to cope and manage their symptoms effectively. As a result, it is crucial to consider the potential impact of surveillance technologies on those with vulnerable mental health profiles [29].

Paranoia and Anxiety

Fear of misuse: The potential for security technologies to be misused or to infringe upon an individual's rights can lead to heightened anxiety and fear. Individuals may be concerned that the data collected or the monitoring conducted by these technologies might be exploited for purposes beyond security, such as unauthorized surveillance, data breaches, or privacy infringements. This fear of misuse can generate a constant sense of unease, with individuals worrying about the potential negative consequences of these technologies. The pervasive fear of misuse can erode trust in institutions and technology providers, leading to a heightened state of anxiety about the impact of security measures on one's personal life and freedoms [30].

Unintended consequences: Individuals may worry about the consequences of security measures, such as false alarms, misidentification, or profiling based on personal characteristics. For instance, a security alarm system that frequently triggers false alarms can lead to frustration and anxiety, as individuals may worry about the disruptions and inconvenience caused by these unintended consequences. Similarly, concerns about being misidentified or profiled based on personal characteristics, such as gender, race, or ethnicity, can create a sense of vulnerability and mistrust. These unintended consequences can lead to a heightened sense of insecurity and stress as individuals grapple with the uncertainty of how they may be affected by the security measures meant to protect them [31].

Sense of vulnerability: The perception of vulnerabilities, whether real or imagined, can contribute to heightened paranoia and anxiety. Individuals may feel exposed and powerless in the face of pervasive security technologies. Even if they have not personally experienced any adverse effects, the constant awareness of potential vulnerabilities can create a state of hyper-vigilance and mistrust. This heightened sense of vulnerability can significantly impact one's mental well-being, as it may lead to a state of constant alertness and apprehension. The sense of vulnerability can erode the feeling of safety and security, essential for mental health and well-being [32].

Ethical Concerns

Erosion of trust: Ethical concerns surrounding the misuse of surveillance data and potential violations of privacy rights can lead to the erosion of trust in institutions and authorities. When individuals perceive that security technologies are being used in ways that infringe upon their rights or are unethical, it can foster skepticism and anxiety about the intentions of those in power. The erosion of trust in institutions can result in mistrust in societal structures, contributing to uncertainty and insecurity. This lack of trust can extend beyond the realm of security technologies and impact individuals' overall mental well-being, as it may lead to a heightened sense of powerlessness and concern about the potential misuse of authority [33].

Moral dilemmas: Using security technologies can present individuals with moral dilemmas, particularly in cases where the ethical implications of these technologies are ambiguous or contentious. For example, facial recognition technology raises ethical questions about its use in law enforcement and public spaces. Individuals may grapple with internal conflict and distress when they are faced with decisions related to the use of such technologies. This internal conflict can lead to anxiety, as individuals may be torn between concerns for public safety and the protection of personal privacy or civil liberties. Moral dilemmas can contribute to unease and ethical anxiety [34].

Civil liberties: Ethical concerns related to the infringement upon civil liberties can evoke distress and anxiety, especially in individuals who highly value personal freedoms and rights. The knowledge that security technologies may encroach on these fundamental liberties can lead to vulnerability and anxiety. Individuals may fear that their civil liberties are at risk and living in a society where their rights are gradually eroding. This concern can result in feelings of powerlessness and an increased sense of insecurity. The protection of civil liberties is an essential component of a just and free society, and ethical concerns related to their potential infringement can have a profound impact on an individual's mental well-being [35].

The role of user perception

How People Perceive Security Technologies

Cultural and societal factors: Cultural norms and societal values have a profound influence on how individuals perceive security technologies. Different cultures and societies may exhibit varying acceptance or resistance to surveillance and security measures. For some, these technologies may be viewed as essential tools for ensuring safety, while for others, they may be seen as invasive and a threat to personal freedoms. The cultural and societal context in which individuals are situated can strongly influence their comfort levels with security technologies. When individuals feel that these technologies align with their cultural and societal values, they are more likely to experience a sense of security and well-being. Conversely, when there is a misalignment between their values and the prevalent societal norms regarding security technologies, this can lead to feelings of discomfort, stress, or anxiety [36].

Past experiences: Personal experiences with security technologies can significantly shape how individuals perceive these measures. Positive experiences, such as when security technologies successfully prevent a security breach or ensure personal safety, can foster a sense of security and trust in these systems. Conversely, negative experiences, such as instances of privacy breaches or misuse of surveillance, can lead to skepticism and anxiety. When individuals have personally encountered issues related to the misuse or malfunction of security technologies, it can result in a heightened sense of vulnerability and mistrust. Past experiences can linger in an individual's memory, affecting their overall mental well-being and influencing their perception of these technologies in the future [37].

Media influence: The media plays a substantial role in shaping public perception of security technologies. Media coverage of security breaches, surveillance misuse, or invasive security measures can heighten concerns and influence public sentiment. News reports or fictional portrayals of the negative consequences of security technologies can lead individuals to question the benefits of these measures and focus on potential drawbacks. Such media influence can contribute to insecurity and anxiety as individuals become increasingly aware of the potential risks and ethical concerns associated with security technologies. Conversely, the media can also highlight the positive aspects and successes of these technologies, which may contribute to a greater sense of well-being and security. How the media frames these technologies can significantly affect the mental well-being of individuals and their perception of security measures [38].

The Psychological Factors at Play

Risk perception: The way people perceive the risks and benefits associated with security technologies can have a significant impact on their mental health and their overall perception of these technologies. When individuals have a heightened perception of the risks involved, they may experience increased anxiety and mistrust. This heightened perception of risk can stem from concerns about potential privacy breaches, misuse of surveillance data, or the invasive nature of security technologies. Such concerns can contribute to a persistent state of unease and uncertainty. Conversely, when individuals feel a sense of security and believe that these technologies effectively mitigate risks, they are more likely to experience reduced stress and an increased sense of well-being. Understanding and managing risk perception is pivotal in influencing individuals' mental health in the context of security technologies [39].

Perceived control: The extent to which individuals feel they have control over their data and privacy is a crucial factor influencing their mental health in the era of security technologies. A lack of perceived control over one's personal information and digital privacy can lead to feelings of vulnerability and stress. When individuals believe that their data is being collected and used without their consent or knowledge, they may feel that their privacy is being violated and their autonomy is being compromised. This sense of vulnerability can create heightened stress and anxiety. In contrast, when individuals have control over their digital data and privacy, they experience greater mental well-being. They feel they can make informed decisions about what information to share and with whom, reducing the anxiety associated with potential privacy violations [40].

Perceived norms: Social norms and peer influence can significantly affect how individuals perceive security technologies and make decisions about their use. Conforming to perceived norms, even when these norms are rooted in misinformation or fear, can impact mental well-being. If individuals perceive that their peers or society significantly view security technologies in a certain way, they may feel pressured to conform to those opinions. This can lead to cognitive dissonance and emotional distress when their beliefs or experiences conflict with these perceived norms. Additionally, misinformation or unfounded fears perpetuated by social norms can lead to unnecessary anxiety or a false sense of security. The influence of perceived norms on security technology perception highlights the importance of informed decision-making and critical thinking in managing the potential impact on mental well-being [41].

Trust and Mistrust in Security Technologies

Trust: Trust in security technologies is a crucial factor associated with feelings of safety and well-being. When individuals trust that these technologies are deployed responsibly, ethically, and in ways that prioritize their safety and privacy, they are more likely to experience a sense of security and lower stress levels. Trust is often built on transparent policies, adherence to ethical guidelines, and a track record of responsible use. When individuals trust that their data is protected, their privacy is respected, and security measures are well-managed, they can navigate the digital and physical world with a greater sense of confidence and peace of mind. Trust in security technologies contributes to overall mental well-being, reducing the anxiety and uncertainty associated with potential risks [42].

Mistrust: Mistrust in security technologies, on the other hand, can lead to heightened anxiety and distress. Individuals may harbor concerns about the potential misuse of these technologies, data breaches, or infringements on civil liberties. Mistrust often emerges from a lack of transparency, perceived ethical violations, or incidents that erode confidence in the responsible use of security measures. When mistrust prevails, individuals may constantly worry about the implications of these technologies on their privacy, personal freedom, and safety. This mistrust can be a significant source of stress and anxiety, affecting overall mental well-being [43].

Balancing trust and mistrust: Achieving a balance between trust and mistrust is essential for maintaining both security and mental well-being. This balance involves ensuring that security technologies are used responsibly and ethically while addressing concerns contributing to mistrust. Responsible use of these technologies, transparent policies, and clear communication about how data is collected, stored, and used can help foster trust. Ethical guidelines and regulations can also play a role in ensuring responsible deployment. At the same time, acknowledging and addressing legitimate concerns and providing avenues for redress can help alleviate mistrust. Achieving this balance requires a collaborative effort between individuals, technology providers, and policymakers to create an environment where security technologies enhance safety without compromising mental well-being [44].

Mitigating negative impacts

Ethical Guidelines and Regulations

Privacy regulations: Stringent privacy regulations and laws, such as the General Data Protection Regulation (GDPR) in Europe, play a vital role in mitigating the negative impacts of security technologies on mental well-being. These regulations set clear boundaries for the collection, storage, and use of personal data, reducing the potential for privacy invasion and associated stress. By requiring organizations to obtain explicit consent for data collection, ensuring data security, and providing individuals with rights to access and control their data, privacy regulations empower individuals and protect their privacy. The knowledge that their data is subject to legal safeguards can contribute to a greater sense of security and well-being, as individuals can trust that their rights are upheld [45].

Ethical frameworks: Organizations and institutions can adopt ethical frameworks prioritizing individual rights and well-being regarding security technologies. These frameworks guide the development and deployment of these technologies with a focus on responsible use. Ethical considerations, such as transparency, consent, and data minimization, can shape the design and implementation of security measures. Ensuring that ethical guidelines are followed can reduce the likelihood of misuse and ethical concerns that may contribute to mistrust and anxiety. Organizations that commit to ethical frameworks demonstrate their dedication to the well-being and privacy of individuals [46].

Transparency and accountability: Transparency in the operation of security technologies, along with mechanisms for accountability, is essential for mitigating negative impacts on mental well-being. Users should be informed about data collection, storage, and sharing practices, as well as the purposes for which their data is used. Institutions and organizations deploying security technologies should provide clear, accessible information about these practices. Additionally, mechanisms for accountability should be established to hold institutions responsible for any breaches of trust or privacy violations. When individuals know that their data is handled transparently and that there are consequences for unethical behavior, they are more likely to trust the technology and the entities responsible for its operation. This trust can contribute to a sense of security and reduce the anxiety associated with potential misuse [47].

User Education and Awareness

Awareness campaigns: Public awareness campaigns play a crucial role in informing individuals about the potential impacts of security technologies, both positive and negative. These campaigns can encompass various media channels, such as television, social media, and community workshops, to disseminate information about cyber threats, privacy concerns, and best practices for online safety. By raising awareness, individuals are better equipped to understand the implications of their digital actions, enabling them to make informed decisions and express any apprehensions. Additionally, awareness campaigns can encourage a more proactive approach to security, prompting individuals to adopt measures safeguarding their digital presence [48].

Digital literacy: Promoting digital literacy involves educating users about various aspects of technology, including data privacy, online security, and the significance of safeguarding personal information. This approach aims to equip users with the knowledge and skills to navigate the digital landscape confidently. By enhancing digital literacy, individuals can develop a deeper understanding of potential cyber threats and the measures required to mitigate risks effectively. This, in turn, can help alleviate the anxiety associated with online vulnerabilities and data breaches, fostering a more resilient and well-informed digital community [49].

Empowerment: Education is a powerful tool for empowering users to take charge of online activities and digital security. By providing individuals with the necessary knowledge and resources, they can better understand the importance of maintaining control over their digital presence and protecting their privacy. Empowered users are more likely to engage with technology in ways that align with their values and concerns, thus fostering a sense of autonomy and confidence in their digital interactions. This empowerment can lead to the development of responsible digital citizens who actively contribute to creating a safer and more secure online environment for themselves and others [21].

Technological Advancements for Privacy Protection

Privacy-enhancing technologies (PETs): PETs are a crucial component of ensuring that security technologies do not infringe upon individuals' privacy rights. These technologies are designed to strike a balance between effective security measures and the protection of personal data. Differential privacy and homomorphic encryption, for example, can be applied to data collection and analysis without exposing sensitive information. Differential privacy adds noise to data in a way that preserves statistical properties while making it difficult to identify individuals. Homomorphic encryption allows computations on encrypted data without decrypting it, enhancing security. By integrating PETs into security solutions, organizations and individuals can benefit from robust security measures without compromising privacy. This can help alleviate concerns about data breaches and unauthorized access to personal information [50].

Anonymization techniques: Anonymization techniques are crucial in ensuring that data collected by security technologies is used responsibly and ethically. These techniques involve removing or altering personally identifiable information from datasets, making it challenging to trace data back to specific individuals. By implementing robust anonymization methods, organizations can mitigate concerns about data misuse, breaches, and unauthorized tracking. Anonymization helps build trust among users, as they are more likely to engage with security technologies when assured that their privacy is respected [51].

User-centric solutions: Designing security technologies with a user-centric approach is pivotal in addressing user concerns and reducing feelings of vulnerability. User-centric solutions prioritize user control over their data and personal settings, allowing individuals to make informed choices about the information they share and how it is used. These solutions may include clear and user-friendly privacy settings, transparent data usage policies, and mechanisms for users to customize their security preferences. When users feel they have control over their online presence and can easily configure their security settings, it enhances their confidence and trust in digital technologies. This, in turn, fosters a sense of empowerment and reduces the fear of being overwhelmed or exploited by these technologies [52].

Future directions

Emerging Security Technologies and Their Potential Impact

Biometric authentication: Biometric authentication methods, such as facial recognition and fingerprint scanning, offer convenience and security benefits. However, they also raise concerns related to privacy and surveillance. Future research should explore the mental health implications of widespread biometric technologies. Users may experience heightened anxiety, fear of identity theft, and concerns about constant surveillance. It is essential to understand how adopting biometrics affects individuals' psychological well-being and whether it leads to feelings of insecurity or invasiveness [53].

Artificial intelligence (AI) and machine learning: The integration of AI and machine learning in security technologies, including predictive policing and threat detection, has the potential to impact individuals' well-being in several ways. These AI-driven systems can introduce bias, discrimination, and concerns about transparency and accountability. Future research should examine the psychological and social consequences of AI-powered security technologies, including how they might affect trust in authorities, feelings of fairness, and concerns about algorithmic decision-making [54].

Internet of things (IoT) security: The rapid expansion of the IoT introduces various security vulnerabilities in connected devices. These vulnerabilities can lead to privacy breaches, unauthorized access, and potential individual harm. Future research should focus on assessing the psychological consequences of IoT-related security issues. Individuals may experience anxiety, fear, and a sense of vulnerability when they realize their connected devices can be compromised. Understanding how these security concerns affect individuals' well-being is crucial to developing effective IoT security measures and supporting users in navigating the IoT landscape safely [55].

Research Gaps and Areas Needing Further Investigation

Long-term effects: While much research has focused on the short-term effects of security technologies, understanding their long-term impact is crucial. Longitudinal studies, which follow individuals over an extended period, are needed to assess how prolonged exposure to these technologies influences mental well-being and privacy concerns. This research can shed light on whether individuals become desensitized to security measures over time, experience heightened stress or anxiety, or adapt positively to improved security practices [56].

Cross-cultural studies: The perception and reaction to security technologies vary significantly across cultural contexts. Conducting cross-cultural studies is vital to understanding these variations and ensuring that security technologies are not one-size-fits-all solutions. Different societies may have distinct values, privacy norms, and attitudes toward technology. Comparative research can help identify cultural factors that influence individuals' mental well-being in the context of security technologies, leading to more culturally sensitive approaches [57].

User-centric research: Research should adopt a user-centric approach by actively involving individuals in the design and evaluation of security technologies. This approach ensures that security solutions align with users' values and concerns, making them more acceptable and effective. Users should have a say in how their data is collected, stored, and used. Involving individuals in the design and decision-making processes can help build trust, reduce feelings of vulnerability, and empower users to take control of their digital security. It also fosters a sense of ownership and responsibility in users [52].

Potential Solutions and Improvements

Ethical-by-design approaches: Developing security technologies with ethics and individual well-being as core principles from the outset is a proactive strategy. It means considering not only security but also the potential ethical and psychological impacts on users. By incorporating ethical considerations into the design process, security technologies can aim to prevent or mitigate negative consequences such as privacy infringements, discrimination, or unintended psychological effects. Ethical-by-design approaches promote responsible innovation and ensure that technologies align with societal values [58].

Privacy-preserving defaults: Designing security technologies with strong privacy-preserving defaults is a user-centric approach. It reduces the burden on users to configure complex settings and make informed choices. When technologies are privacy-oriented by default, they provide a higher level of protection from the start, minimizing the risk of unintentional data exposure. Users can then opt-in to share additional data if they choose to do so, promoting a sense of control and trust in the technology [59].

Regulatory frameworks: Governments and regulatory bodies are crucial in ensuring that security technologies balance security and individual rights. The latest research findings should inform these frameworks to adapt to evolving technologies and emerging concerns. Effective regulations can set standards for responsible data handling, privacy protection, and user rights. They provide legal mechanisms to hold organizations accountable for breaches and unethical practices, reinforcing the importance of individual well-being and privacy [60].

User empowerment tools: Empowering users to control their digital presence and data is essential. Tools and features like simplified privacy settings and data management interfaces make it easier for individuals to understand and manage their information. This control fosters a sense of agency, enabling users to make decisions aligned with their preferences and comfort levels. When users feel more in control, they are more likely to engage with technology in ways that support their well-being and privacy [24].

Public awareness initiatives: Continued public awareness campaigns and educational efforts are necessary to inform individuals about the potential impacts of security technologies. These initiatives empower users to make informed choices and express their concerns. When people are aware of the implications of their online actions, they can take steps to protect their privacy, understand potential risks, and advocate for responsible technology use. Public awareness campaigns create a more informed and vigilant user base [61].

Interdisciplinary collaboration: Collaboration among technologists, social scientists, ethicists, and policymakers is crucial for developing holistic solutions. Security technologies have broad societal implications, and interdisciplinary collaboration can help consider these wider consequences. By bringing together expertise from various fields, it's possible to design technologies that are not only secure but also ethically sound and aligned with user well-being [62].

## Conclusions

In conclusion, examining the impact of security technologies on mental health reveals a multifaceted relationship that demands careful consideration. This comprehensive review has uncovered the positive and negative implications of these technologies, emphasizing their capacity to enhance personal safety and reduce fear while underscoring concerns regarding privacy invasion, surveillance-related stress, and ethical dilemmas. User perception, trust, and risk perception play pivotal roles in shaping the mental health outcomes associated with security technologies. Striking a balance between the necessity of security and preserving individual well-being is a moral and practical imperative. To achieve this equilibrium, it is essential to prioritize ethical guidelines, user-centric solutions, interdisciplinary collaboration, and public awareness initiatives. As security technologies evolve, ongoing research, ethical considerations, and user empowerment are pivotal in fostering a harmonious coexistence between security and mental health in our interconnected world.

## References

[1] Borky JM, Bradley TH (2018). Protecting information with cybersecurity. Effective Model-Based Systems Engineering.

[2] Alawida M, Omolara AE, Abiodun OI, Al-Rajab M (2022). A deeper look into cybersecurity issues in the wake of Covid-19: a survey. J King Saud Univ Comput Inf Sci.

[3] Anastasi G, Bambi S (2023). Utilization and effects of security technologies in mental health: a scoping review. Int J Ment Health Nurs.

[4] Bauer M, Glenn T, Monteith S, Bauer R, Whybrow PC, Geddes J (2017). Ethical perspectives on recommending digital technology for patients with mental illness. Int J Bipolar Disord.

[5] (2023). Chapter 5-Protecting Your System: Physical Security. https://nces.ed.gov/pubs98/safetech/chapter5.asp.

[6] Jang-Jaccard J, Nepal S (2014). A survey of emerging threats in cybersecurity. J Comput Syst Sci.

[7] Insider I (2023). Role of CCTV cameras : Public, privacy and protection. https://www.ifsecglobal.com/video-surveillance/role-cctv-cameras-public-privacy-protection/.

[8] (2023). Physical security access control systems | Greetly. https://www.greetly.com/blog/physical-security-access-control-systems.

[9] Fontes C, Hohma E, Corrigan CC, Lütge C (2022). AI-powered public surveillance systems: why we (might) need them and how we want them. Technol Soc.

[10] (2023). Mental health. https://www.who.int/news-room/fact-sheets/detail/mental-health-strengthening-our-response.

[11] Umberson D, Montez JK (2010). Social relationships and health: a flashpoint for health policy. J Health Soc Behav.

[12] Pavitra KS, Chandrashekar CR, Choudhury P (2007). Creativity and mental health: a profile of writers and musicians. Indian J Psychiatry.

[13] Mariotti A (2015). The effects of chronic stress on health: new insights into the molecular mechanisms of brain-body communication. Future Sci OA.

[14] Rajgopal T (2010). Mental well-being at the workplace. Indian J Occup Environ Med.

[15] (2023). What role do security systems and surveillance play in loss prevention strategies. https://www.waffleinsurance.com/resources/insuranpedia/what-role-do-security-systems-and-surveillance-play-in-loss-prevention-strategies.

[16] (2023). What is incident response? A complete guide. https://www.techtarget.com/searchsecurity/definition/incident-response.

[17] Socha R, Kogut B (2020). Urban video surveillance as a tool to improve security in public spaces. Sustainability.

[18] (2023). What is a home security system and how does it work?. https://www.security.org/home-security-systems/what-is-a-home-security-system/#:~:text=A%20home%20security%20system%20is,ADT%20review%2C%20or%20Vivint%20review.

[19] (2023). A comprehensive overview of safety and security in the workplace. https://www.getkisi.com/resources/safety-security-workplace-guides.

[20] Darling-Hammond L, Flook L, Cook-Harvey C, Barron B, Osher D (2020). Implications for educational practice of the science of learning and development. Appl Dev Sci.

[21] Dwivedi YK, Kshetri N, Hughes L (2023). Opinion paper: “So what if ChatGPT wrote it?” Multidisciplinary perspectives on opportunities, challenges and implications of generative conversational AI for research, practice and policy. Int J Inf Manage.

[22] (2023). What is cybersecurity & importance of cyber security. https://www.simplilearn.com/tutorials/cyber-security-tutorial/what-is-cyber-security.

[23] Dwivedi YK, Hughes L, Baabdullah AM (2022). Metaverse beyond the hype: multidisciplinary perspectives on emerging challenges, opportunities, and agenda for research, practice and policy. Int J Inf Manage.

[24] Quach S, Thaichon P, Martin KD, Weaven S, Palmatier RW (2022). Digital technologies: tensions in privacy and data. J Acad Mark Sci.

[25] Li Y, Liu Q (2021). A comprehensive review study of cyber-attacks and cyber security; emerging trends and recent developments. Energy Rep.

[26] Jain AK, Sahoo SR, Kaubiyal J (2021). Online social networks security and privacy: comprehensive review and analysis. Complex Intell Syst.

[27] Goodman FR, Kelso KC, Wiernik BM, Kashdan TB (2021). Social comparisons and social anxiety in daily life: an experience-sampling approach. J Abnorm Psychol.

[28] Platform EL (2023). What Is Self-Censorship? How Does It Kill Media Freedom?. https://www.liberties.eu/en/stories/self-censorship/43569.

[29] Makwana N (2019). Disaster and its impact on mental health: a narrative review. J Family Med Prim Care.

[30] Institute of Medicine (1994). Confidentiality and Privacy of Personal Data. Health Data in the Information Age: Use, Disclosure, and Privacy.

[31] (2023). What is a false alarm?. https://www.isarsoft.com/knowledge-hub/false-alarm.

[32] Geiger S, Galasso I, Hangel N, Lucivero F, Watts G (2023). Vulnerability and response-ability in the pandemic marketplace: developing an ethic of care for provisioning in crisis. J Bus Ethics.

[33] Kisselburgh L, Beever J (2022). The ethics of privacy in research and design: principles, practices, and potential. Modern Socio-Technical Perspectives on Privacy.

[34] Saheb T (2023). "Ethically contentious aspects of artificial intelligence surveillance: a social science perspective". AI Ethics.

[35] Nagel T (1995). Personal rights and public space. Philos Public Aff.

[36] National Academies of Sciences, Engineering Engineering, and Medicine (2018). Addressing the Social and Cultural Norms that Underlie the Acceptance of Violence: Proceedings of a Workshop—In Brief. Addressing the Social and Cultural Norms that Underlie the Acceptance of Violence: Proceedings of a Workshop—In Brief.

[37] Khando K, Gao S, Islam SM, Salman A (2021). Enhancing employees information security awareness in private and public organisations: a systematic literature review. Comput Secur.

[38] Pulido CM, Ruiz-Eugenio L, Redondo-Sama G, Villarejo-Carballido B (2020). A new application of social impact in social media for overcoming fake news in health. Int J Environ Res Public Health.

[39] Naslund JA, Bondre A, Torous J, Aschbrenner KA (2020). Social media and mental health: benefits, risks, and opportunities for research and practice. J Technol Behav Sci.

[40] Bondre A, Pathare S, Naslund JA (2021). Protecting mental health data privacy in India: the case of data linkage with Aadhaar. Glob Health Sci Pract.

[41] Pedersen ER, Miles JN, Hunter SB, Osilla KC, Ewing BA, D'Amico EJ (2013). Perceived norms moderate the association between mental health symptoms and drinking outcomes among at-risk adolescents. J Stud Alcohol Drugs.

[42] Thiebes S, Lins S, Sunyaev A (2021). Trustworthy artificial intelligence. Electron Mark.

[43] Rodriguez LM, DiBello AM, Øverup CS, Neighbors C (2015). The price of distrust: trust, anxious attachment, jealousy, and partner abuse. Partner Abuse.

[44] Aldboush HHH, Ferdous M (2023). Building trust in fintech: an analysis of ethical and privacy considerations in the intersection of big data, ai, and customer trust. Int J Financ Stud.

[45] Yuan B, Li J (2019). The policy effect of the general data protection regulation (GDPR) on the digital public health sector in the European union: an empirical investigation. Int J Environ Res Public Health.

[46] (2023). Ethics of Artificial Intelligence | UNESCO. https://www.unesco.org/en/artificial-intelligence/recommendation-ethics.

[47] Felzmann H, Villaronga EF, Lutz C, Tamò-Larrieux A (2019). Transparency you can trust: transparency requirements for artificial intelligence between legal norms and contextual concerns. Big Data Soc.

[48] Dwivedi YK, Ismagilova E, Hughes DL (2021). Setting the future of digital and social media marketing research: perspectives and research propositions. Int J Inf Manage.

[49] Team L (2023). Importance of Digital Literacy Skills for Students. https://www.learning.com/blog/reasons-digital-literacy-is-important-for-students/.

[50] Jha D (2023). What are Privacy Enhancing Technologies? Why are They Important?. https://www.lightbeam.ai/post/what-are-privacy-enhancing-technologies-why-are-they-important.

[51] (2023). Data Anonymization. Corporate Finance Institute. https://corporatefinanceinstitute.com/resources/business-intelligence/data-anonymization/.

[52] Grobler M, Gaire R, Nepal S (2021). Usage and usability: redefining human centric cyber security. Front Big Data.

[53] National Research Council (US) Whither Biometrics Committee (2010). Cultural, social, and legal considerations. In: biometric recognition: challenges and opportunities. Cultural, Social, and Legal Considerations. In: Biometric Recognition: Challenges and Opportunities.

[54] Xu Y, Liu X, Cao X (2021). Artificial intelligence: a powerful paradigm for scientific research. Innovation (Camb).

[55] Taherdoost H (2023). Security and internet of things: benefits, challenges, and future perspectives. Electronics.

[56] Faden VB, Day NL, Windle M (2004). Collecting longitudinal data through childhood, adolescence, and young adulthood: methodological challenges. Alcohol Clin Exp Res.

[57] Li Y (2022). Cross-cultural privacy differences. Modern Socio-Technical Perspectives on Privacy.

[58] Stahl BC, Antoniou J, Ryan M, Macnish K, Jiya T (2022). Organisational responses to the ethical issues of artificial intelligence. AI Soc.

[59] Bi R, Chen Q, Chen L, Xiong J, Wu D (2020). A privacy-preserving personalized service framework through Bayesian game in social IoT. Wirel Commun Mob Comput.

[60] Kavanagh C (2023). New Tech, New Threats, and New Governance Challenges: An Opportunity to Craft Smarter Responses? Carnegie Endowment for International Peace. https://carnegieendowment.org/2019/08/28/new-tech-new-threats-and-new-governance-challenges-opportunity-to-craft-smarter-responses-pub-79736..

[61] Seymour J (2018). The impact of public health awareness campaigns on the awareness and quality of palliative care. J Palliat Med.

[62] Jacobs N, Brewer S, Craigon PJ (2021). Considering the ethical implications of digital collaboration in the food sector. Patterns (N Y).

